# Pharmacogenomics cascade testing (PhaCT): a novel approach for preemptive pharmacogenomics testing to optimize medication therapy

**DOI:** 10.1038/s41397-020-00182-9

**Published:** 2020-08-25

**Authors:** Don Roosan, Angela Hwang, Moom R. Roosan

**Affiliations:** 1 Department of Pharmacy Practice and Administration, College of Pharmacy, Western University of Health Sciences, Pomona, CA USA; 2 Department of Pharmacy Practice, School of Pharmacy, Chapman University, School of Pharmacy, Irvine, CA USA

**Keywords:** Public health, Translational research, Genetics research

## Abstract

The implementation of pharmacogenomics (PGx) has come a long way since the dawn of utilizing pharmacogenomic data in clinical patient care. However, the potential benefits of sharing PGx results have yet to be explored. In this paper, we explore the willingness of patients to share PGx results, as well as the inclusion of family medication history in identifying potential family members for pharmacogenomics cascade testing (PhaCT). The genetic similarities in families allow for identifying potential gene variants prior to official preemptive testing. Once a candidate patient is determined, PhaCT can be initiated. PhaCT recognizes that further cascade testing throughout a family can serve to improve precision medicine. In order to make PhaCT feasible, we propose a novel shareable HIPAA-compliant informatics platform that will enable patients to manage not only their own test results and medications but also those of their family members. The informatics platform will be an external genomics system with capabilities to integrate with patients’ electronic health records. Patients will be given the tools to provide information to and work with clinicians in identifying family members for PhaCT through this platform. Offering patients the tools to share PGx results with their family members for preemptive testing could be the key to empowering patients. Clinicians can utilize PhaCT to potentially improve medication adherence, which may consequently help to distribute the burden of health management between patients, family members, providers, and payers.

## Introduction

Over the last two decades, the incorporation of pharmacogenomics (PGx) into routine clinical practice has been steadily increasing [1, 2]. Resources such as PharmGKB, the Clinical Pharmacogenetics Implementation Consortium (CPIC), and the Dutch Pharmacogenetics Working Group (DPWG) have been advancing the translation of complex PGx information into actionable phenotypes that are useful for clinicians. More recently, the Electronic Medical Records and Genomics (eMERGE) and the Implementing Genomics in Practice (IGNITE) networks have focused on embedding genomics data with electronic health records (EHR) [3]. While programs such as eMERGE and IGNITE have made strides in the use of PGx results in clinical practice, there are still barriers to the widespread utilization of PGx. For example, not many EHRs support discrete PGx data that can be translated to Clinical Decision Support (CDS), and many leading EHR vendors impose an extra fee to support PGx modules. Moreover, reimbursement for PGx testing is still done on a case-by-case basis by most insurance companies. For example, Medicare will only pay for CYP2D6 and CYP2C19 PGx testing for specific drugs or disease conditions, while private health insurance payers often vary in their willingness to pay for PGx testing. Among other financial challenges, the additional costs and inaccessibility of PGx data further demotivate healthcare systems and providers to implement PGx testing. Fortunately, providers, payers, and patients understand the impact and importance of PGx [4]. For instance, the annual burden of medication non-adherence is estimated to be $300 million [5]. In contrast, the validation of the effectiveness of medications by PGx incentivizes patients towards improved medication adherence [6]. From the perspective of the payer, more health insurance companies are realizing the financial benefits of PGx testing, which decreases unnecessary utilization and costs, and are thus reimbursing for PGx tests [7]. Furthermore, patients have a desire to manage and share PGx results with family members. Unlike tests such as bloodwork, which need to be repeated, germline DNA does not change over the course of a person’s life, and thus the long-term benefits of PGx results are applicable over the lifetime of the patient. While research has focused on EHR PGx integration within health systems, little has been done to facilitate the sharing of PGx results between family members and clinicians [8–10].

### Current models for sharing PGx data

**Fig. 1:**
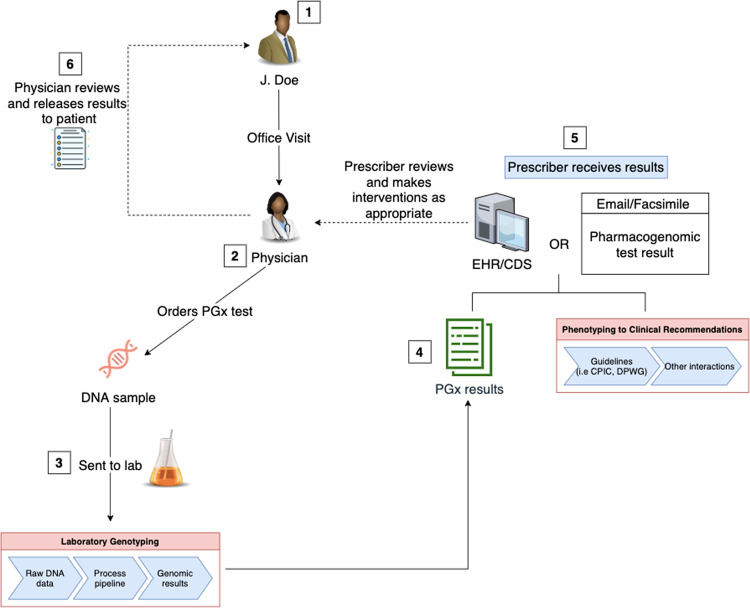
Current typical workflow of a clinical pharmacogenomics (PGx) test from ordering to reviewing. First, a PGx test is ordered by the clinician. Second, the laboratory receives the physician’s order and sends a test kit to the patient to collect the patient’s DNA sample. The laboratory processes the patient’s sample after collection, and the raw genotyping data is processed. Third, the raw genotyping results are converted into a human-readable format and sent to the prescriber via electronic health records (EHR), email, or fax. Lastly, the results are shared with the patient by the physician.

Direct-to-consumer (DTC) PGx tests often bypass the review of a clinician and send results to patients directly, and patients are in control of sharing their results with clinicians. However, DTC PGx tests are marred by concerns regarding test validity, quality, misinterpretation, and the potential for inappropriate medical action. In contrast, the analytical validity and accuracy of clinical PGx tests are usually very robust (accuracy > 99%), and they are often Clinical Laboratory Improvemennt Amendments (CLIA)-certified or FDA-cleared. Figure 1 illustrates the workflow of how patients typically receive clinical PGx test results at present. First, the clinician assesses the patient’s need for a PGx test and, if deemed necessary, orders the test. After the laboratory receives the physician’s order, a test kit is sent to the patient to collect the patient’s DNA sample. Currently, most laboratories send PGx results as a plain text representation of the interpretation report and transmit the results to patients and providers using Health Level 7 (HL7) v2 standard or a simple static pdf file [1]. Unfortunately, static pdf files often become buried in emails or locked inside EHRs and are thus not readily available to the patient. Moreover, if a patient was tested for multiple pharmacogenes, the resulting multipage report is often too extensive for the provider to interpret without CDS.

### Preferences for sharing results

Although results may not be pertinent at the time of testing, PGx tests have long-term implications. A recent study involving 869 patients showed that 87% of patients understood their PGx results and recognized the implications for the future usage of PGx [11]. Patients with a higher level of understanding elected to share results with providers for the most optimum care in their health-decision making [12–15]. After sharing results with providers, several studies show that the majority of patients were satisfied with the conversations they had with their health care providers (HCPs) and felt that the PGx results could be utilized to improve their health [6, 16]. In addition, recent studies have reported that most patients are willing to not only share test results with providers but also with family members (Table 1) [6, 11, 15–17]. Patients who shared their PGx data with family members believed that the results could be significant in improving the health of their family members, while family members indicated that they desired to receive genetic test results when they were relevant and appreciated the information [18–20]. By sharing PGx results with family members, patients can shed light on heritable instances of drug toxicity or treatment failures that family members experienced in the past.

**Table 1:** Willingness of patients to share pharmacogenomics (PGx) test results with family members and their health care providers (HCPs).

Are patients willing to manage PGx results? (Yes/No)	Number of participants	Findings	Rationale	Authors [Ref.]
Yes	1464	909 (62%) planned to share genomic results with HCPs	To improve health	van der Wouden et al. [12]
Yes	17	11 (65%) reported that they will share PGx results with physicians, 8 (47%) shared results with family	To select the best treatment option	Haga et al. [6]
Yes	588	406 (69%) planned to share PGx results with HCPs, family, children, siblings, friends, parents, and pharmacists	Results would be important for health care decisions and medication selection or family’s health	Olson et al. [11]
Yes	478	430 (90%) indicated that they were extremely comfortable sharing and managing PGx results	Wanted doctors to manage care and avoid duplicate testing	Haga et al. [14]
Yes	54	34 (63%) reported discussing PGx results with provider and 43 (80%) discussed results with family	To help in health care decision making	Lemke et al. [16]
Yes	77	56 (72.7%) reported sharing results from BRCA1/2 or Lynch syndrome testing with HCPs and 58 (75.3%) with family members	To inform family about risks, receive recommendations from HCPs	Taber et al. [15]
Yes	62	All participants wanted to share results with family and 37 (60%) with providers, while 20 (31%) wished to withhold PGx information from providers	Patients (*n* = 20, 31%) who did not want to share with providers claimed that providers were too busy, not interested, or incompetent	Madadi et al. [22]
Yes	319	249 (78%) viewed and navigated electronic health records but 70 (22%) patients did not want to see results	Patients (*n* = 70, 22%) who did not want to see PGx results expressed concerns for confidentiality, computer security, lack of interest, fear of results, etc.	Pyper et al. [21]

A small number of studies—which included patients who were willing to share results—also reported that some patients did not want to share test results with providers owing to concerns about computer confidentiality and illiteracy or provider disinterest [21–23]. Patients also indicated that they preferred a phone or in-person conversation for explanations of results [11]. In recent years, providers have had increasing amounts of exposure to PGx and have been trained to make clinical decisions based on PGx results. In addition, studies have found that patients preferred to receive web-based resources in tandem with a lengthy conversation about PGx test results [15, 23, 24]. Moreover, issues related to privacy and HIPAA have been improved with new technological platforms. For example, platforms such as Amazon Web Services (AWS) and G-suite by Google provide cloud-based services that are both HIPAA compliant and secured. Thanks to growing technological advancements and the prevalence of smartphones, patients can now easily access and view test results via user-friendly mobile applications.

### Sharing family pharmacogenomics data

Family history has long been used in clinical practice to assess risk for many diseases (e.g., diabetes, cardiovascular diseases). Additionally, family history has also been utilized to determine if further screening is necessary for various cancers, such as breast or colorectal cancers. Recent evidence supports the investigation of the responses of first-degree relatives to medications for patients with the same disease [25]. For example, functional CYP2C19 activity is required for the activation of clopidogrel in order to inhibit platelet aggregation. If a patient has a history of recurrent myocardial infarction or stroke post stent-placement despite being on clopidogrel, it would be prudent to order PGx testing to rule out the suboptimal CYP2C19 enzyme activity. If the patient exhibits a poor metabolite phenotype, their siblings and/or children would likely exhibit at least an intermediate CYP2C19 phenotype. Other medications that are metabolized by CYP2C19 may also require dose adjustment or alternatives based on the CYP2C19 phenotype. Citalopram, escitalopram, tricyclic antidepressants, voriconazole, and proton pump inhibitors are few of the drugs that are metabolized by CYP2C19 and would likely require such adjustments. Instead of genetically testing every family member, the PGx data of one family member could inherently be applied to members of the family who present a similar medical scenario [25].

### Pharmacogenomic cascade testing (PhaCT)

Cascade testing is a method that has been used to genetically test for characterized diseases such as familial hypercholesterolemia, cancer, and certain arrhythmias [20, 26–28]. In genetic testing for cancer, the traceback method has been used to identify mutations in cancer patients who have not been genetically tested. When a mutation is found through traceback, a cascade testing is then performed on at-risk family members [28]. Tests are initially performed in a cascading fashion using first-degree relatives owing to genetic similarities or overlap [20]. Cascade testing provides patients that have a family history of cancer with a confirmation of the presence of allelic variations similar to that of the diagnosed family members, and thus an opportunity for patients to act proactively on their results. Therefore, traceback testing uses a reactive approach of screening for putative mutations when the diagnosis already exists in a patient, while cascade testing prospectively uses family history data to identify mutations preemptively among the at-risk family members.

The idea of cascade testing can be implemented in PGx testing. However, cascade testing is not currently used in PGx testing. The information from a patient’s PGx results can help family members that may be concerned about similar medication interactions to pursue a preemptive PGx testing. For example, in Gennis et al., three siblings out of 12 developed phenytoin hypersensitivity reactions [29]. An in vitro study of cells from the patients who exhibited hypersensitivity reactions showed an increase in toxicity from metabolites of phenytoin and carbamazepine. Four other siblings from the same family who never took anticonvulsants also exhibited a high degree of toxicity from phenytoin and carbamazepine metabolites in vitro. This family highlights the inherited nature of phenytoin hypersensitivity and its importance as a candidate for PGx cascade testing. If one of the siblings had received PGx results confirming the presence of HLA-B*15:02 or CYP2C9-atypical metabolism—which predisposes them to Stevens-Johnsons Syndrome (SJS), Toxic Epidermal Necrolysis Syndrome (TENS), or other side effects—the results would also be highly pertinent to the rest of the family members, as well as their children. While PGx testing was not widely available at the time this study was conducted, this family is a prime example of how an entire family could have benefitted from the pharmacogenomics cascade testing (PhaCT) method, which would have effectively identified a history of sensitivity reactions to certain antiepileptic medications. As PGx becomes more widely accessible, it is important that patients share results with family members in order to effectively identify individuals that are at risk for adverse drug events or medication failures. Also, with the increasing complexity of patients’ data from discrete sources, it is imperative to integrate family PGx data to optimize therapy outcomes for family members [30–33].

### The future of integrated PhaCT

The Translational Pharmacogenetics Program (TPP) of the National Institutes of Health (NIH) Pharmacogenomics Research Network has discussed the diversity and variability in ordering, storing, and representing PGx results. Although PGx data standards are offered by SNOMED CT (Systemized Nomenclature of Human and Veterinary Medicine, Clinical Terms), LOINC (Logical Observation Identifiers Names and Codes), and Health Level Seven International (HL7 v2, v3Clinical Document Architectures, FHIR), the standards are not routinely implemented by PGx labs or EHRs [34]. This lack of data standardization in PGx results is a significant barrier to sharing results between different healthcare systems, patients, and other providers. Health Information Exchange (HIE) allows patient information to be accessed throughout different levels of care using an interoperable system. Although HIE initiatives have been progressing since the HITECH Act in 2009, most HIE occurs across healthcare systems and does not extend to patients [35]. In contrast, sharing PGx data is an aspect of HIE that would include patients in the accountability of their health. Allowing patients to share PGx results with their family members for preemptive testing in a HIPAA-protected environment could be the key to engaging and empowering patients. Ultimately, it is necessary to develop PGx data in a structured, scalable, and EHR-agnostic shareable format from various third-party laboratories. This discrete model for PGx data will allow patients to easily share PGx results with family members and use the results for future generations as well.

**Fig. 2:**
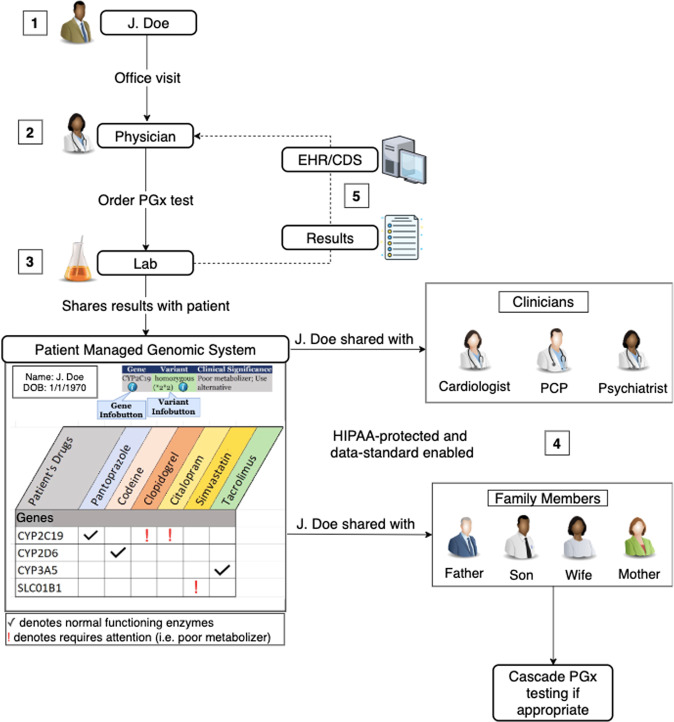
A proposed integrative model for operationalizing pharmacogenomics data sharing and pharmacogenomic cascade testing (PhaCT). In this model, the ordering of the test, the collection of the patient’s sample, and the translation of genomics data into clinically actionable results will remain the same. However, once the results are finalized, they will be sent to an ancillary patient-managed genomic system (PMGS). Within the PMGS system, patients will have access to the results and will be able to share results with other clinicians or family members. Lastly, the PMGS could also have the ability to send the results to the electronic health record (EHR), which could then be sent to the prescribing physician.

In the current typical workflow of a PGx test (Fig. 1), patients may view their results either as a pdf or other static text format. An ancillary genomics system, capable of storing and sharing discrete, computable data, has the potential to improve the shareability of PGx results with their family members, current and future providers. We propose a potential model for sharing PGx results, which is illustrated in Fig. 2. Once a PGx test is ordered by a prescriber through either the EHR or a point-of-care web-based platform, the lab sends the machine-readable data to an ancillary patient-managed genomic system (PMGS), which would be created for patients to review and manage their results. The PMGS will also store a human-readable version of the clinically actionable PGx results. PGx test results can be accessed both by a mobile or web application, as well as the EHR. Within the PMGS system, patients will have access to the results and can share results with other clinicians or family members. Once shared by the patient, the computable results in the EHR may employ CDS logic and show actionable recommendations to the appropriate clinicians. In this model, both patients and providers have access to the same results. Ultimately, patients are able to share these results with family members, as well as with other providers who might be part of a different medical group. As a result, the patient is the gatekeeper of the PGx results, and therefore is in control of sharing them.

This model will only be successful if visual and data analytics are integrated into the design of the user platform [36, 37]. A user-friendly design would encourage patients to check PGx results easily whenever new medications are being considered for their treatment plan. While previous studies have investigated ideal visual and data analytics platforms on the provider side, future research is needed to investigate patient preferences and usability [36, 38]. With an effective design, patients will be more motivated to share and utilize results to improve decision making regarding their health care in collaboration with their providers in order to foster shared decision making.

One of the obstacles to implementing shared decision making is engaging patients at every level of care [39]. However, by allowing patients to share results with family members and providers, patients are actively involved at all levels of health care decisions. Another benefit of patient participation and a shareable platform is the potential to decrease polypharmacy and complexity regarding medications [31, 36]. When patients have PGx results that are easily accessible, patients will be able to utilize this tool to check medications with providers at the point-of-care in order to prevent duplications in therapy or unnecessary or overly complicated therapy modalities. Using this model, patients will fully understand the purpose of each medication as well as their treatment plan [11]. In addition, since family members are aware of medications that are being taken within the family, they can hold each other accountable and share the responsibility of health management.

There is also a cause for concern regarding the ethical and legal implications of sharing results with family members [28]. Fortunately, with laws in place to protect patients against genetic discrimination via the Genetic Information Nondiscrimination Act (GINA) and often stricter state-specific GINA protections, patients can be at peace with sharing genomic information [19]. With a shareable platform, patients will have the ability to decide how much information to share with family members and for how long. Currently, no laws are in place to prevent patients from disclosing such information [19]. Most importantly, sharing PGx results will be at the discretion of the patient. However, physicians should encourage at-risk patients to inform family members who could benefit from earlier interventions [40].

## Conclusions

By incorporating family medication history along with PGx data, both HCPs and patients will be equipped with more knowledge to determine the most appropriate therapy. More specifically, an advanced CDS system that can leverage the shared family PGx data may have the capability to adequately support providers and patients in shared decision-making [30, 41]. As discussed above, patients indicate that they are not only willing to share results with providers but with family members as well. Unfortunately, there are currently barriers to the integration of family history with PGx data as well as to allowing patients to share results with family. One example of these barriers is the lack of security and privacy in sharing results with others. However, recent advancements in technology can address this issue by letting patients choose precisely what to share and with whom to share it.

In the future, the use of family history information and PGx data have the potential to improve patient clinical outcomes by incorporating PhaCT at a national level. By integrating PhaCT, patients can rely on the PMGS to ascertain whether a family member requires preemptive PGx testing. PhaCT can have a significant positive impact on getting insurance reimbursement for PGx testing. Once providers review family members’ results, the case for ordering a PGx test can be substantial to save the patient from potentially similar adverse drug reactions previously experienced by others in the family. Thus, insurance companies may have a stronger motivation to reimburse for PGx testing to reduce overall healthcare costs. By sharing results, patients are integrated into their own health care experience, as well as that of their family. Future studies are needed to explore the features of PMGS with user-friendly visual analytics for PGx data sharing.

## Supplementary information

Presentation of PhaCT model
